# Supplementary material for: Resolving the full spectrum of human genome variation using Linked-Reads
Source: Genome Res. 2019 Apr;29(4):635–45. doi: 10.1101/gr.234443.118 (PMC6442396; doi:10.1101/gr.234443.118)
Supplement: Supplemental Material [file supp_gr.234443.118_Supplemental_longranger-2.2.zip › longranger-2.2.tar.bz2!/lr2.2-public/lib/stan/cmdstan/stan/doc/api/html/structstan_1_1gm_1_1program.html]

Stan: stan::gm::program Struct Reference


|  |  |
| --- | --- |
|  | Stan  2.5.0  probability, sampling & optimization |


- Main Page
- Related Pages
- Namespaces
- Classes
- Files

- Class List
- Class Index
- Class Hierarchy
- Class Members

All Classes Namespaces Files Functions Variables Typedefs Enumerations Enumerator Friends Macros Pages

- stan
- gm
- program

Public Member Functions |
Public Attributes |
List of all members

stan::gm::program Struct Reference

`#include <ast.hpp>`

|  |  |
| --- | --- |
| Public Member Functions | |
|  | program () |
|  | |
|  | program (const std::vector< function\_decl\_def > &function\_decl\_defs, const std::vector< var\_decl > &data\_decl, const std::pair< std::vector< var\_decl >, std::vector< statement > > &derived\_data\_decl, const std::vector< var\_decl > &parameter\_decl, const std::pair< std::vector< var\_decl >, std::vector< statement > > &derived\_decl, const statement &st, const std::pair< std::vector< var\_decl >, std::vector< statement > > &generated\_decl) |
|  | |

|  |  |
| --- | --- |
| Public Attributes | |
| std::vector< function\_decl\_def > | function\_decl\_defs\_ |
|  | |
| std::vector< var\_decl > | data\_decl\_ |
|  | |
| std::pair< std::vector  < var\_decl >, std::vector  < statement > > | derived\_data\_decl\_ |
|  | |
| std::vector< var\_decl > | parameter\_decl\_ |
|  | |
| std::pair< std::vector  < var\_decl >, std::vector  < statement > > | derived\_decl\_ |
|  | |
| statement | statement\_ |
|  | |
| std::pair< std::vector  < var\_decl >, std::vector  < statement > > | generated\_decl\_ |
|  | |

## Detailed Description

Definition at line 795 of file ast.hpp.

## Constructor & Destructor Documentation

|  |  |  |  |  |
| --- | --- | --- | --- | --- |
| stan::gm::program::program | ( |  | ) |  |

Definition at line 1341 of file ast\_def.cpp.

|  |  |  |  |
| --- | --- | --- | --- |
| stan::gm::program::program | ( | const std::vector< function\_decl\_def > & | *function\_decl\_defs*, |
|  |  | const std::vector< var\_decl > & | *data\_decl*, |
|  |  | const std::pair< std::vector< var\_decl >, std::vector< statement > > & | *derived\_data\_decl*, |
|  |  | const std::vector< var\_decl > & | *parameter\_decl*, |
|  |  | const std::pair< std::vector< var\_decl >, std::vector< statement > > & | *derived\_decl*, |
|  |  | const statement & | *st*, |
|  |  | const std::pair< std::vector< var\_decl >, std::vector< statement > > & | *generated\_decl* |
|  | ) |  |  |

Definition at line 1342 of file ast\_def.cpp.

## Member Data Documentation

std::vector<var\_decl> stan::gm::program::data\_decl\_

Definition at line 797 of file ast.hpp.

std::pair<std::vector<var\_decl>,std::vector<statement> > stan::gm::program::derived\_data\_decl\_

Definition at line 799 of file ast.hpp.

std::pair<std::vector<var\_decl>,std::vector<statement> > stan::gm::program::derived\_decl\_

Definition at line 802 of file ast.hpp.

std::vector<function\_decl\_def> stan::gm::program::function\_decl\_defs\_

Definition at line 796 of file ast.hpp.

std::pair<std::vector<var\_decl>,std::vector<statement> > stan::gm::program::generated\_decl\_

Definition at line 804 of file ast.hpp.

std::vector<var\_decl> stan::gm::program::parameter\_decl\_

Definition at line 800 of file ast.hpp.

statement stan::gm::program::statement\_

Definition at line 803 of file ast.hpp.

---

The documentation for this struct was generated from the following files:

- src/stan/gm/ast.hpp
- src/stan/gm/ast\_def.cpp


---

[ Stan Home Page ]

*© 2011–2014,
Stan Development Team.*
